# The Aesthetic Self. The Importance of Aesthetic Taste in Music and Art for Our Perceived Identity

**DOI:** 10.3389/fpsyg.2020.577703

**Published:** 2021-03-09

**Authors:** Joerg Fingerhut, Javier Gomez-Lavin, Claudia Winklmayr, Jesse J. Prinz

**Affiliations:** ^1^Berlin School of Mind and Brain, Humboldt-Universität zu Berlin, Berlin, Germany; ^2^Department of Philosophy, The University of Pennsylvania, Philadelphia, PA, United States; ^3^Max-Planck-Institute for Mathematics in the Sciences, Leipzig, Germany; ^4^The Graduate Center, CUNY, New York, NY, United States

**Keywords:** aesthetic emotions, aesthetic preferences, art, beauty, moral values, music, personal identity, self

## Abstract

To what extent do aesthetic taste and our interest in the arts constitute who we are? In this paper, we present a series of empirical findings that suggest an *Aesthetic Self Effect* supporting the claim that our aesthetic engagements are a central component of our identity. Counterfactual changes in aesthetic preferences, for example, moving from liking classical music to liking pop, are perceived as altering us as a person. The Aesthetic Self Effect is as strong as the impact of moral changes, such as altering political partisanship or religious orientation, and significantly stronger than for other categories of taste, such as food preferences (Study 1). Using a multidimensional scaling technique to map perceived aesthetic similarities among musical genres, we determined that aesthetic distances between genres correlate highly with the perceived difference in identity (Study 2). Further studies generalize the Aesthetic Self Effect beyond the musical domain: general changes in visual art preferences, for example from more traditional to abstract art, also elicited a strong Self Effect (Study 3). Exploring the breadth of this effect we also found an *Anaesthetic Self Effect.* That is, hypothetical changes from aesthetic indifference to caring about music, art, or beauty are judged to have a significant impact on identity. This effect on identity is stronger for aesthetic fields compared to leisure activities, such as hiking or playing video games (Study 4). Across our studies, the Anaesthetic Self Effect turns out to be stronger than the Aesthetic Self Effect. Taken together, we found evidence for a link between aesthetics and identity: we are aesthetic selves. When our tastes in music and the arts or our aesthetic interests change we take these to be transformative changes.

## Introduction

Within philosophy, psychology, and neuroscience, research on personal identity has tended to focus on certain traits such as memory or experience of agency. In ordinary life, however, the identities we construct and convey to others are often related to our preferences: the things we care about and like. Recently, this aspect of identity has been studied with a focus on moral values. A number of studies have suggested that changes in moral values are construed as changes in personal identity (Strohminger and Nichols, 2014; Prinz and Nichols, 2016; Heiphetz et al., 2017). But what about other kinds of values? Our daily life is replete with decisions that reflect our aesthetic values and preferences. What shall I wear? What shall I watch on television? What music shall I listen to? Some follow their favorite musician on social media and adapt further aesthetic preferences from them. Others invest time and significant resources to see art shows in museums. Our hypothesis in this paper is that aesthetic preferences and the arts could be also closely linked to the self and that a change in taste and aesthetic values may be construed as a change in who one is.

Philosophers of art have investigated that nature of taste and aesthetic judgment (Hume, 2000, [1739]; Zangwill, 2019), but there has been comparatively little work on the links between taste and identity. Here we explore this relationship with four experimental studies. We show that changes in taste are interpreted as changes in identity and we found that the degree of impact on identity varies with the degree of the aesthetic change. The self has many dimensions, but one seems to relate to the arts. As such, our findings suggest that the construction of identity includes our relationship to cultural artifacts. That bears on how we should think about the self — it may be more cultural than some theories have emphasized — and how we think about the arts — they are not merely forms of entertainment but also constitute important values we care about and that are central to us. Hence, we are aesthetic selves: central aspects of our identity are constituted by cultural and art-related preferences.

### Prior Work Relating to Art, Aesthetics, and Identity

To our knowledge, there has been no work to date using psychological methods to explore intuitions about the relationship between aesthetic taste, the arts, and personal identity. Still, there is research that adds plausibility to our hypothesis.

First, there is much work in sociology on the nature of subcultures. Philosophers and cognitive scientists tend to focus on highly individual aspects of identity, such as autobiographical memories, but, for sociologists and some social psychologists, identity is more frequently associated with the social groups to which we belong, and these include groups united by taste in the arts. The most obvious examples come from music: youth subcultures such as goths, punks, and deadheads are conceptualized as contributing to identity (Hebdige, 1979). Sociologists have also traced connections between aesthetic taste and social class. For example, preference for classical (Katz-Gerro, 2002) or eclectic music (Peterson and Kern, 1996) might help one indicate one’s social status. Similar things hold for art expertise and museum attendance (Bourdieu et al., 1990; Hanquinet, 2013). Such observations indicate that our taste can help us form social groups and signal to others who we are and where we stand in society. Related to this is the idea of aesthetic tribalism: Taste choices come in groups. If we share one aesthetic commitment with a social group we might unwittingly adopt more of their choices, aesthetic and otherwise, picking up its aesthetic convictions rather than consciously adopting them, which gives our aesthetic participation a highly social touch (Lopes, 2018). Interestingly, seminal research on “minimal group” formation also has used taste in paintings (Klee vs. Kandinsky) to induce preferential treatment of in-groups (Tajfel, 1970).

Second, there has been a substantial body of work examining links between personality traits and aesthetic interests for music and the arts. Studies into art preferences – the extent to which individuals like or dislike different styles of paintings – have represented the dominant approach in the area of personality and art, no doubt because of the relative straightforwardness of classifying artistic products according to established schools. Even before personality traits were “invented” (i.e., prior to the development of trait taxonomies), psychological eminencies such as Burt (1933) and Eysenck (1940) examined personality differences in ratings of different paintings. Even today, empirical aesthetics is in no small part shaped by individual differences in taste and preference (Jacobsen, 2006). Factors such as “openness to experience” as well as other traits can be used to predict artistic preferences (Furnham and Walker, 2001; Chamorro-Premuzic et al., 2009; Swami and Furnham, 2014). Significantly less research has been devoted to the questions of how much aesthetic traits actually matter to us and to what extent we perceive them as being central to us as a person. There have been some claims regarding an “artistic personality” whose openness, curiosity, imagination, and creativity leads to a greater proclivity for aesthetic experiences (Chamorro-Premuzic et al., 2007). Yet the complementary question has, to our knowledge, never been studied: would a change in my preferences change me?

Third, there is recent evidence that moral values play a central role for our identity, prompting researchers to postulate a “moral self” (Strohminger and Nichols, 2014; Prinz and Nichols, 2016). Within philosophy, morals and aesthetics are regarded as the two main domains of value. Unlike “descriptive” domains, which capture how things actually are, normative domains describe how things should be, and terms of evaluation (such as good and bad) are used to assess cases that meet or violate those norms. In both morals and aesthetics, we make such evaluative judgments. This invites the question: If morals are important to identity, why not aesthetics? The link between morality and identity may relate to the fact that morality is emotionally charged (Greene and Haidt, 2002). We feel our moral values deeply, and experience intense emotions when they are instantiated or violated (Avramova and Inbar, 2013). The same might be true for aesthetic values. That we invest a lot of energy and resources to engage in aesthetic experiences of artworks or to encounter our favorite musicians and bands in concerts is already an indication that our aesthetic choices might in fact matter a great deal to us. It only seems plausible that our aesthetic taste is also important to who we are. It is this taste that determines the range of aesthetic objects and experiences we value. Similar to our moral evaluations of social situations aesthetic evaluations are also inherently affective. They are motivational states related to dispositions of our embodied and situated self to act on the world (Prinz, 2011; Fingerhut and Prinz, 2018a). This link of art and emotion (complementary to the moral-emotions link) provides a further reason to explore the possibility that aesthetic taste is related to identity.

These findings offer circumstantial reasons for hypothesizing an “aesthetic self.” If preferences for certain arts are linked to social group membership, personality, and emotionally grounded values, then changes in our aesthetic preferences should be perceived as a threat to our identity and transform us. The main aim of this investigation is, therefore, to explore whether changes in aesthetic tastes actually exert an impact on perceived identity.

### Self and Diachronic Personal Identity

Philosophical discussions of personal identity usually focus on “diachronic identity,” or changes across transformations that take place in time. Philosophers also distinguish between qualitative and quantitative (or “numerical”) concepts of identity. To be numerically identical with oneself in the past is to be the exact same person. Thought experiments suggest that this notion of identity is often not what we care about. For example, if a person walked into a fission machine that created two psychological and physical duplicates of herself, both these duplicates would feel a sense of identity with the original person, but they couldn’t be numerically identical to her (Parfit, 1984). What we care about is some kind of continuity with our past, be it physical or psychological. In ordinary life, when we think about being the same person or same self over time, metaphysical notions of numerical identity matter less than preserving certain traits we see as centrally belonging to us.

When it comes to preservation of identity, it is likely that we care about more than just one trait. There are numerous theories of the self each presenting a different perspective and utilizing an array of methodologies. Some emphasize physical continuity (Williams, 1970) while others focus on preservation of psychological traits (Parfit, 1971), such as memory (Locke, 1690), personal narratives (Schechtman, 1996), or agency (Korsgaard, 1989). Within embodied cognition the autonomous organism has been identified as the basis for continuity from a first person perspective (Fuchs, 2017), combining a biological with a psychological approach.

These are sometimes presented as competing theories, but they need not be. Recent “pattern theories of the self” aim to unify and systemize those theories by pointing toward a clusters of features that constitute the self (Gallagher, 2013; Newen, 2018). Such a theory requires a multifaceted approach. It defends such clusters against prevalent deflationist and reductionist accounts that highlight only one feature at the expense of others as being central for a self. We also follow them in their call for an interdisciplinary approach that aims to organize claims ranging from aspects of minimal embodiment and self-consciousness (including their neural dynamics), affect (such as emotional patterns), cognition (such as memory), to more situated aspects (such as cultural and normative practices). In particular, we will contribute to the understanding of the latter aspect by focusing on aesthetic engagement with the arts and cultural artifacts (such as music and visual art) by possibly touching on the affective basis of those relations as well. We also follow pattern theories in another aspect: instead of identifying minimal, necessary conditions we focus on what could be considered jointly sufficient conditions for an individual self. The idea here is that several factors, albeit to varying extents, contribute to the construction of such a self.

In this respect we will explore changes in one’s life under which we might not consider ourselves to be the same person after the change. What changes would make somebody a new person? What kind of transitions in traits, preferences, and activities would have the biggest impact on who we think we are? Which changes are viewed as transformational with respect to identity?

### Testing Intuitions About Identity

Only recently have traits thought to be integral to sustaining one’s identity been tested with experimental methods. In a pioneering experimental philosophy study, Nichols and Bruno (2010) found that both memory and preservation of the body are judged to be important for personal identity. As noted above, more recent work, has explored the role of moral values in identity, and preservation of these is judged to be even more important for identity than memory (Strohminger and Nichols, 2014; Prinz and Nichols, 2016). In a typical study, participants are asked to consider situations in which various traits change, such as memory, cognitive capacities, and moral values, and then are asked to judge whether the affected individual is the same person after the change. Moral change vignettes have proven to elicit a significantly greater impact than any other trait to which it has been compared to date.

We aim to extend this empirical literature by more systematically addressing the category of taste, aesthetic and otherwise. As mentioned above, aesthetics constitutes a further domain of values, beyond morals. In psychology, values are often seen as norms that guide our behavior and are captured in series of multidimensional scales (Hanel et al., 2018). Our investigation builds upon this research and extends it toward aesthetic values (such as beauty, the arts, and preference for specific artforms and music). Curiously, prior studies found that changes in preferences (loss of enjoyment of favorite food or rock music) and changes in art engagement (loss of the ability to appreciate art) failed to yield a strong impact on our perceived identity (Strohminger and Nichols, 2014). Given the literature reviewed above, we felt this relationship demands a more thorough investigation. In a series of experiments, we presented participants with vignettes that prompt them to imagine their tastes changing and then asked them to what extent they would perceive themselves to be the same person after the change. The striking finding across all our experiments is that taste changes are among the changes that present the biggest threat to the identity of a person. We therefore could show that we are not just moral selves but crucially aesthetic selves as well.

## Study 1: a Series of Worldly Changes

### Introduction

Our first study examines the impact of a series of mundane life changes on the perceived identity of a person. We adapted methods employed in previous vignette studies that specified a trait change and asked participants to what degree they would perceive themselves or others as being the same person after such a change (Blok et al., 2005; Strohminger and Nichols, 2014). Whereas those studies often focus on an extraordinary event (car crash, brain transplant, and incarnation) followed by a psychological trait change (loss of memory and moral change) we simplified our conditions by including everyday changes that might occur in one’s life while omitting the reference to a mediating variable (such as an extraordinary event or an underlying neurodegenerative cause, see Strohminger and Nichols, 2015). Our vignettes included changes in neighborhood, occupation, leisure activities, political and religious orientation, and most importantly taste. Each participant was introduced to the concept of personal identity in the following way:^1^

For the presented example of a change, we want you to ask yourself: “Would I be the same person?” if this change takes place. How would I regard myself after the change? And how would others regard me?”

This was followed by a question regarding the importance of a domain for the participant (e.g., music, politics, religion, etc.), followed by a vignette from this domain, e.g.:

Suppose your taste in music changed dramatically. For example, if you enjoy only classical music, imagine you grew to like listening to only pop music.

Or:

Suppose your religion changed dramatically. For example, if you are a person of faith, imagine you became an atheist.

Participants were then asked to what extent they would consider themselves the same person after the change on a scale from “completely” to “not at all.” This is what we considered a judgment of identity. The higher a participant’s rating on this scale the more we take it that a given trait has an effect on perceived identity. Hence, when we speak of a larger Self Effect, it is because participants’ ratings on this scale were comparably higher than for other traits.

We were particularly interested in assessing how changes in taste impact the sense of self compared to changes in hobbies or changes in moral domains. We therefore included two taste conditions (a change in music preference and a change in food taste from “liking spicy to liking only mild food”) and two changes in leisure activities (“becoming passionate about playing video games” or “hiking” after having no interest in those activities before). In line with the recent literature on the topic, moral changes should have a bigger impact than other changes (Prinz and Nichols, 2016). We therefore included two conditions that should indicate a moral value change, for which we predicted the largest Self Effect. Those were changes in political orientation (conservative to more left wing) and changes in general religious outlook (from atheist to believer).

We predicted that changes in musical taste would impact identity, though perhaps not as strongly as changes in moral values (exemplified by the two highly moralized domains religion and politics). Contrary to previous studies that found that among a variety of conditions changes in aesthetic preferences and art engagements were among the changes with the least impact on identity (Strohminger and Nichols, 2014), we hypothesized that taste in the arts (e.g., musical taste) should be associated with identity. This was based on the literature reviewed above. We had no such clear predictions with respect to changes in taste outside the arts, such as food preferences, although it has been argued that the homeostatic need to appraise food might also be the basis for aesthetic appraisals of objects more generally and supervenes on some shared brain circuitry (Brown et al., 2011). In order to test whether the presented changes were indeed perceived as changes in morals or in taste, we assessed this in a pretest with an independent sample (consult Pretest 1 below).

Aside from leisure activities (video games and hiking) we included two more comparison categories: occupation (changes with respect to job or chores), and location (change of neighborhood or country). We predicted that changes in each of these dimensions would have some impact on perceived identity. In particular, we expected to replicate the impact of moral change on identity, and our key prediction was that aesthetic changes (explored via the category of taste) would also be highly associated with changes in identity—a prediction that has not been established or systematically explored in prior work.

### Materials and Methods

#### Pretest 1

251 German adults (*Mdn* age = 34, range 18–68; 36.9% identified as female, 61.1% identified as male) were recruited for the Pretest online via *Clickworker*. Seven additional participants were excluded for failing to complete the study. Approval for all studies was obtained from the CUNY University Committee on Activities Involving Human Subjects (UCAIHS; IRB-2016-0794).

This test was set up in order to assess whether our grouping of traits into a Moral and Taste change category was valid (consult Table 1 for our initial grouping). We exclusively focused on ratings on moral and taste change in our Pretest, since those two domains comprise what are considered values in philosophy. We therefore conducted a Pretest with an independent sample from the main study. Participants were randomly assigned to one condition producing a 10 × 1 between-subjects design. We asked participants to rate how important a domain was for them (e.g., music, politics, religion, neighborhood, etc.) on a seven-point Likert scale anchored at “not at all” and “very much.” This was followed by a vignette indicating a change in that domain. We then asked participants to rate whether this amounts to a change in moral values (Q1: “To what extent is this change a change in moral values?”) or in taste (Q2: “To what extent is this change a change in taste?”) on a seven-point Likert scale anchored at “not at all” and “very much.” Q1 and Q2 were presented in random order. Afterward, participants were directed to answer a small series of demographic questions (Supplementary Material A2).

**TABLE 1:** List of vignettes used in Study 1.

**Category**	**Domain**	**Item**
Morals	Religion	Being a faithful believer to becoming an atheist
	Politics	Changing from liberal to conservative party
Taste	Music	Liking classical music to liking pop music
	Food	Liking spicy food to liking mild food
Leisure	Recreation	Not caring about hiking to growing passionate about it
	Pastime	Not playing video games to becoming passionate about them
Occupation	Job	Working as a lawyer to becoming a teacher
	Chores	Hating housework to enjoy vacuuming
Location	Neighborhood	Moving from inner city to the suburbs
	Country	Moving from Germany to southern hemisphere

*Ordered per category in descending order of predicted impact on the self.*

#### Study 1

359 German adults (age = *17.3%: 18–24, 38.7%: 25–35, 30.1%: 35–50, 13.9%:*>*50;* 45.4% identified as female, 54.3% as male) were recruited online via *Amazon’s MTurk* platform. Eight additional participants were excluded for failing to complete the study.

The set-up and wording were the same as in the Pretest (10 × 1 between-subjects design): we asked participants to rate how important a certain domain was for them and presented them one change vignette that corresponded to this domain. Then they had to rate to what extent they would consider themselves to be the same after the change (“Would you regard yourself as the same person?”) on a seven-point Likert scale from 1 (“very much”) to 7 (“not at all”), followed by the same demographic questions as in Pretest 1.

### Results and Discussion

#### Pretest 1

In this test we could confirm our coding for taste and moral change. When asked to rate to what extent the changes we presented constitute a moral (Q1) or taste change (Q2), religion and politics were rated highest on the moral change question (Table 2). Food and music ranked highest on the taste change question. Interestingly, our two Leisure conditions (videogames and hiking) ranked highly on both measures. We first tested participant ratings in our ten conditions for measures for normality; however, Kolmogorov–Smirnov and Shapiro–Wilk’s tests showed that participant scores were not normally distributed (all *p* < 0.05).^2^ A Kruskal–Wallis test showed that there was a statistically significant difference across conditions for both Q1 [*H(9)* = 26.351, *p* = 0.002], and Q2 [*H(9)* = 27.639, *p* = 0.001].

**TABLE 2:** Means and SD for amount of moral change (Q1) and taste change (Q2) in Pretest 1.

**Moral change**	**Mean (SD)**	**Taste change**	**Mean (SD)**
Religion	4.72 (1.823)	Food	5.19 (1.494)
Politics	4.44 (1.444)	Music	5.08 (1.269)
Job	4.13 (2.007)	Recreation	4.77 (1.250)
Recreation	3.92 (1.708)	Pastime	4.60 (1.497)
Country	3.60 (1.600)	Country	4.40 (1.697)
Pastime	3.40 (1.414)	Politics	4.20 (1.470)
Neighborhood	3.23 (1.648)	Job	4.04 (1.546)
Food	3.19 (1.545)	Religion	3.96 (2.010)
Music	3.04 (1.698)	Neighborhood	3.81 (1.710)
Chores	2.96 (1.620)	Chores	3.33 (1.795)

*Listed are all domains in descending order of means.*

1. Moral Change: *post hoc* pairwise comparisons for our moral change question were done using Mann–Whitney *U* tests and showed no significant difference between our Religion (median, “Mdn” = 5.0) and politics conditions (*Mdn* = 5.0): *U* = 269.5, *z* = −0.851, *p* = 0.395. Changes in religion and politics were rated significantly higher as a moral change than the Music (*Mdn* = 3.0) and Food conditions (*Mdn* = 3.0). Music vs. Religion: *U* = 163.0, *z* = −3.095, *p* = 0.002, and vs. politics: *U* = 180.5, *z* = −2.776, *p* = 0.005; Food vs. Religion: *U* = 171.0, *z* = −2.94, *p* = 0.003, and vs. Politics: *U* = 184.5, *z* = −2.694, *p* = 0.007.
2. Taste change: our Food (*Mdn* = 5.5) and Music conditions (*Mdn* = 5.0) received the highest participant ratings of taste-change, but did not differ significantly from each other, *U* = 305.5, *z* = −0.613, *p* = 0.54. Our Food condition differed significantly from both Politics (*Mdn* = 4.0), *U* = 198.00, *z* = −2.45, *p* = 0.014, and Religion (*Mdn* = 5.0), *U* = 211.00, *z* = −2.19, *p* = 0.028, whereas Music differed significantly from Politics: *U* = 212.0, *z* = −2.224, *p* = 0.026, but not from Religion: *U* = 239.5, *z* = −1.663, *p* = 0.096. Apart from the last result, the Pretest fully confirmed our coding for the main categories of moral and taste change.

#### Study 1

##### Self Effect Within Categories

We carried out pairwise comparisons of the rated Self Effect within our five categories (Morals, Taste, Leisure, Occupation, and Location), which each consisted of two domains. To compare the Self Effect between two domains within a category we used the non-parametric Mann–Whitney *U* test. We only found one significant difference in those pairwise comparisons. This was within the Taste category: Music taste changes (*Mdn* = 4.0) had a significantly stronger Self Effect than food taste changes (*Mdn* = 2.0), *U* = 194.5, *z* = −2.666, *p* = 0.008. We therefore separated music and food into independent categories: “Taste-Aesthetic” and “Taste-Gustatory,” all other pairs were combined into meta-conditions correlating to a category. This left us with six meta-conditions: Morals, Music, Food, Location, Occupation, and Leisure.

##### Self Effect Between Categories

**FIGURE 1:**
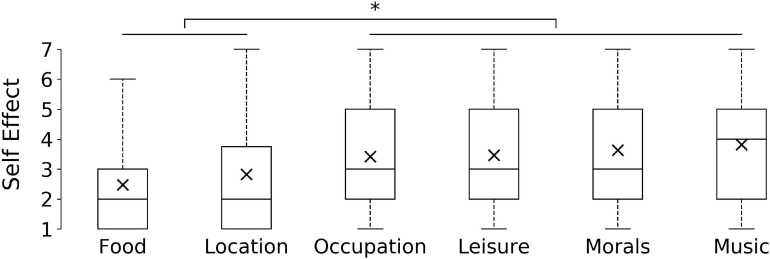
Box plots of participants’ responses for Self Effect across categories in Study 1, ^∗^*p* < 0.05, two-tailed. *Y*-axis indicates full range of possible answers on a seven-point Likert scale from 1 (“very much”) to 7 (“not at all the same person”).

We used Kruskal–Wallis comparisons between our meta-conditions adjusted by Bonferroni corrections for multiple tests, followed by pairwise comparisons using Mann–Whitney *U* test. A Kruskal–Wallis test indicated that our categories elicited an effect on our perceived self-change measure, *H*(5) = 15.132, *p* = 0.01 (For a list of means per items and category consult Table 3). Mann–Whitney *U* tests between our conditions showed that Self Effect did not differ between Location (*Mdn* = 2.0) and Taste-Gustatory conditions (*Mdn* = 2.0), *U* = 734.5, *z* = −0.391, *p* = 0.696. Those two conditions received significantly lower Self Effect ratings compared to all other conditions such as Occupation (*Mdn* = 3.0), Leisure (*Mdn* = 3.0), Morals (*Mdn* = 3.0), and Taste-Aesthetic (*Mdn* = 4.0), all *p* = 0.037. The biggest difference was between the Taste-Gustatory (*Mdn* = 2.0) and the aesthetic vignette referring to a change in music-preference: Taste-Aesthetic (*Mdn* = 4.0), *U* = 194.5, *z* = −2.666, *p* = 0.008 (Figure 1).

**TABLE 3:** Means and SD for Self Effect for all domains in Study 1.

**Category**	**Domain**	**Mean (SD)**	
		**Per item**	**Per category**
Taste-A	Music	3.81 (1.806)	3.81 (1.806)
Morals	Religion	4.12 (2.155)	3.63 (2.023)
	Politics	3.30 (1.856)	
Leisure	Recreation	3.55 (1.955)	3.46 (1.893)
	Pastime	3.37 (1.816)	
Occupation	Job	3.48 (1.815)	3.42 (1.713)
	Chores	3.40 (1.671)	
Location	Country	3.06 (2.154)	2.82 (1.947)
	Neighborhood	2.58 (1.681)	
Taste-G	Food	2.48 (1.446)	2.48 (1.446)

*In descending order of mean per category and then domain per category. Our Taste category has been split, *post hoc*, into two Taste categories: Taste-Aesthetic (Music) and Taste-Gustatory (Food).*

#### Discussion

Our first finding was that the Taste-Aesthetic condition elicited the second strongest Self Effect (3.81). This gave us an initial indication of an Aesthetic Self Effect that had not been reported to date and that contradicts earlier findings that established no such effect for aesthetic conditions (Strohminger and Nichols, 2014). Only the Religion condition exhibited a nominally higher mean for our self-measure (4.12).^3^ Interestingly, none of the two measures of our Pretest (impact on moral values, impact on taste) correlated significantly with the Self Effects for the same ten items rated in Study 1. There is a positive correlation between Moral ratings and Self Effect, though, that simply may have not reached significance due to the low number of correlated items (*n* = 10, Spearman’s correlations of Moral-Pretest and Self Effect: *r* = 0.285, *p* = 0.425). There was no indication of a correlation between Taste and Self Effect (*r* = −0.079, *p* = 0.829), which led us conclude that the Taste dimension we asked for in the Pretest is either in itself not central for identity or contains several dimensions (consult discussion below). This is in line with the finding that Taste-Aesthetic and Taste-Gustatory (ranking both highest on the taste measure Q2) also differ significantly with respect to our self-measure. Because of this finding we conducted a post hoc split of the Taste category into Music and Food and decided to explore this difference in the studies reported below.

That we found stronger Self Effects for the Taste-Aesthetic and Morals categories confirmed our general predictions for value changes. Somewhat surprisingly, the Self Effect elicited in those domains did not differ significantly from our Leisure or Occupation domains (which in part might be explain by a difference in wording, see Study 4 below). The Location category worked well as a control, generating a significantly smaller Self Effect than all other categories (except Taste-Gustatory) and possibly driven by the lack of any strong value change associated with relocation. In another set of studies on the effects of immigration in this respect we indeed were able to show that a relocation that is accompanied by a value change elicits a significantly stronger Self Effect (Gomez-Lavin et al., n.d.).

**FIGURE 2:**
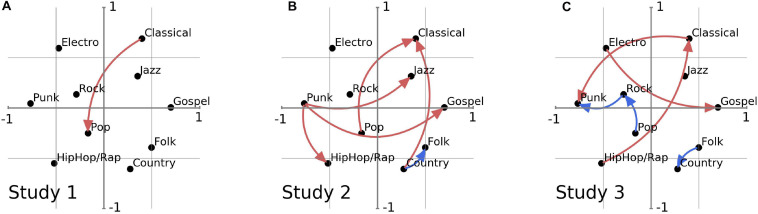
Similarity map of music genres in two-dimensional space as assessed in Pretest 2. The *y*-axis represents dimension 1 of our mapping, the *x*-axis dimension two. Changes in preference that have been used in the respective studies are indicated by an arrow that also marks the direction of change. **(A)** Graph shows Classical-to-Pop change used in Study 1, red indicates that this change is perceived as having a significantly stronger Self Effect than the other taste change in this study, *p* < 0.01, consult also Figure 1. **(B)** Aesthetic taste changes presented in Study 2: Only Country-to-Folk (indicated by blue) shows a significantly lower Self Effect to the other preference changes presented (red), *p* < 0.05. **(C)** Aesthetic Taste Changes presented in Study 3: The Far-Genres (red) show a significantly higher Self Effect compared to the Close-Genres (blue), *p* < 0.05.

Our Pretest confirmed that Music and Food preference changes are perceived as changes in taste, as those conditions ranked highest with respect to our taste-measure (Table 2). While the Pretest supported our grouping, Music and Food differed significantly with respect to our self-measure (Figure 2 and Table 3). One explanation is that the two domains of our initial Taste category might differ with respect to social signaling. Our music preference might be an indicator of the social groups we belong to, which might not hold to the same extent for our food preferences. Another possible confound could be the ambiguity of the Taste question (Q2) itself. The question (“To what extent is this change a change in taste?”) might be perceived as asking two things at the same time: whether there is significant shift in the preferred experiential qualities, *or* whether it is a shift with respect to the more longstanding aesthetic sensitivity of a person that might also be seen as an overall improvement (e.g., used in the context of a person having an enhanced taste for the ‘finer things in life’).^4^ Future studies might aim to disambiguate the two meanings, e.g., by additionally asking whether one might be more or less a “person of taste” after the change.

Based on the initial finding for music preference changes (Taste-Aesthetic), we first wanted to explore the robustness of the Self Effect *within* aesthetic domains (by including further aesthetic change conditions). In order to determine whether we found a genuine aesthetic effect, we also decided to implement more direct measures of aesthetic differences in the follow-up studies. The aim was to assess a possible direct relation of aesthetic contrasts to our Self Effect (see Study 2 and 3 on changes in music genre preferences). So far, we did not find a correlation between what was considered a taste change in Pretest 1 and the self-measure in Study 1. Yet perhaps such a correlation could be found for aesthetic differences of the items used within one vignette, which we explored in Study 2.

## Study 2: Exploring the Aesthetic Self Effect for Music

### Introduction

Study 2 was set-up to replicate our findings from Study 1 and to establish the robustness of the Aesthetic Self Effect for music. We therefore wanted to ensure that the effect was not an artifact of our specific choice of genres in Study 1. After all, we only described one transition (from classical to pop music preference, *n* = 27), and our effect might therefore be contingent upon this particular change.^5^ We decided to first map aesthetic differences between music genres in a Pretest and then, in a second step, to apply our self-measure to conditions that included changes between specific genres of our mapping (Study 2). For the Pretest we asked participants to rate the similarities between all possible combinations of ten genres. We used multidimensional scaling to construct a map of the relationship among musical genres based on participants’ perceptions of their similarities (Figure 2A). The Pop genre turned out to be centrally located in the two-dimensional similarity space, whereas Classical is located in the periphery near Jazz in the upper right-hand quadrant. The further any two genres are located from one another in the space, the less similar they are perceived.

We mapped the genres in order to be able to relate an aesthetic measure of musical similarity to our Self Effect measure. In order to test whether such aesthetic differences had an impact on our identity question, we created a series of variants of our original prompt based on the information we garnered from our multidimensional maps. We decided to test the effect of directionality (by including a reversal of the change from our original vignette in Study 1). Additionally we wanted to explore the possible role of the dimensions of the map (therefore including changes across the *y* and *x*-axes) and whether distance to the center of the map would have an effect (including changes between genres that are at roughly the same distance from the center, whereas our original Classical-to-Pop vignette was more a center-periphery change). As a last condition we also included the two genres that are closest together, Folk and Country. All others genre comparisons used for Study 2 were 2–4 times as distant on the map (consult Figures 2A–C for full graphical rendering of all the changes for which we tested a Self Effect). We predicted that a change from the center of the map to the periphery (e.g., Pop-to-Classical) might elicit a stronger effect because it would indicate a more refined and distinguished taste. We speculated that possibly one of our two dimensions (depicted as the *y* and *x*-axis) might correlate more strongly to changes in perceived identity.^6^

Hence, we predict that changing one’s preference among aesthetically similar genres (such as Country-to-Folk) should yield a smaller Self Effect, thus confirming that the aesthetic properties of genres drive their relative impacts to identity. Another prediction was that some genre changes would signal a change in social group membership related to the respective genres. To test for this possibility, we included an additional dependent variable in Studies 2 (and all follow-up studies) that aimed to measure whether changes in taste were perceived as impacting social relationships. This was added in an effort to determine whether the Aesthetic Self Effect that we found in Study 1 might stem from the fact that musical preferences can be associated with membership in social groups, such as subcultures. The conjecture was that sociality, alongside the aesthetic properties of genres we determine, could drive our effects. As others have shown: preferences for music are likely affected by both social and auditory characteristics (Rentfrow et al., 2011). More generally our findings align with studies that establish that personality traits seem to systematically correlate with music genre preferences (for a summary of studies to this effect see: Schäfer and Mehlhorn, 2017).

### Materials and Methods

#### Pretest 2

45 German adults (*Mdn* age = 38, range 18–60 years 51.1% self-identified female) were recruited for the Pretest online via *Clickworker*. One additional participant was excluded for failing to complete the study.

This test was configured in order to assess the similarity of music genres. Participants were asked to rate ten genres, presented in pairs, for musical similarity yielding 90 permutations of which 45 unique combinations were presented to participants to mitigate participant fatigue. For any two genres, participants were presented with a scale from 1–100 with 1 anchored at “not at all similar” and 100 at “completely similar.” The data were analyzed using the PROXSCAL algorithm in SPSS, with ordinal measurement and Euclidian distance. In addition to our standard demographic questions we included also an authoritarianism scale as well as TIPI in this part of the study (consult Supplementary Material A2).

#### Study 2

364 German adults (*Mdn* age = 34; range 18–70; 50.3% identified as female, 47.3% identified as male) were recruited online via *Clickworker*. Ten additional participants were excluded for failing to complete the study.

The set-up and wording were exactly as in Study 1 (6 × 1 between-subjects design). We generated new vignettes using genres from the Pretest (for the selection of genres see the discussion above). As before, we asked participants how important music was for them and presented them a change-vignette with a taste change from one genre to another. They had to rate to what extent they would consider themselves to be the same after the change. Compared to Study 1 we also added a Relationship question in order to assess whether the perceived change correlated to a social change: “To what extent do you think that such a dramatic change of one person would influence the relationship to their friends?” on a Likert scale anchored from 1 (“not at all”) to 7 (“completely”). Followed by the same demographic questions as above.

### Results and Discussion

#### Pretest 2

Following standard procedures in multidimensional scaling (Hout et al., 2013; Tollefsen et al., 2014) we produced a two-dimensional map of our ten genres with the y-axis representing the first dimension and the x-axis the second dimension (Figure 2). With this multidimensional scaling method, a two-dimensional solution of participant judgments explained 95.78% of the variance in their responses.^7^ (We listed all direct mean rated distance for all our genre combinations as well as the Euclidean distances of those genres in the map in Supplementary Material B).

#### Study 2

Kruskal Wallis tests indicated a significant difference for Self Effect across our music preference changes *H(5)* = 11.953, *p* = 0.035. In particular, all genre changes showed a significant difference from the Country-Folk pair (*Mdn* = 2.0, for the means for all conditions consult Table 4). This was confirmed by Mann–Whitney *U* tests for difference comparing Country-Folk to: Pop-Classic (*Mdn* = 4.0, *U* = 920.0, *z* = −2.446, *p* = 0.014), Punk-Jazz (*Mdn* = 4.0, *U* = 899.5, *z* = −2.944, *p* = 0.004), Country-Classic *(Mdn* = 5.0, *U* = 916.0, *z* = −2.751, *p* = 0.006), Punk-Gospel *(Mdn* = 5.0, *U* = 958.5, *z* = −2.459, *p* = 0.014) and Punk-Hip Hop (*Mdn* = 4.0, *U* = 939.0, *z* = −2.314, *p* = 0.021).

**TABLE 4:** Means and SD for Self Effect for all changes in genre preferences in Study 2.

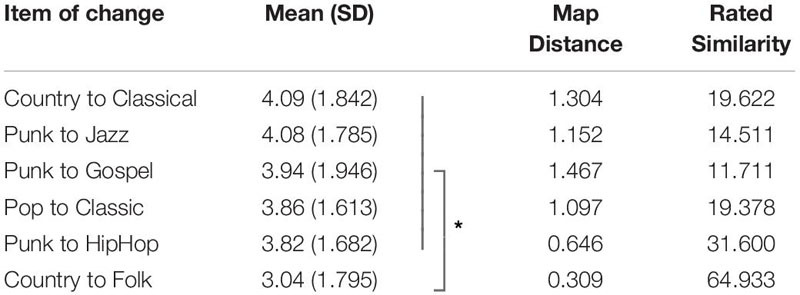

In descending order of mean Self Effect per item, *p < 0.05, two-tailed. Including the means of the Euclidean distance on the map and the mean pairwise rated similarity of the genres from the pretest.

**FIGURE 3:**
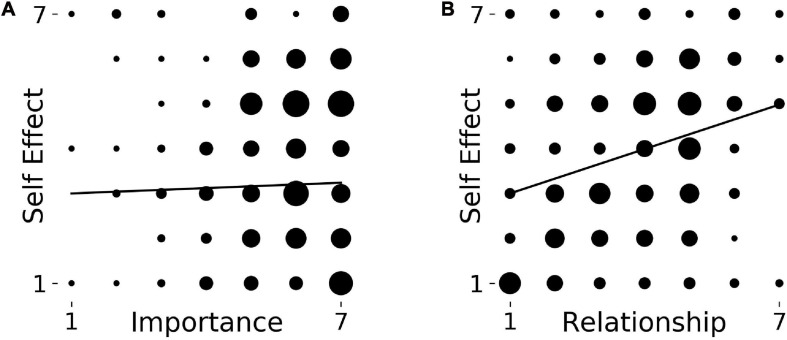
Scatter plots of Spearman’s correlations for within-subject ratings of Study 2: **(A)** shows correlation between rated Self Effect and rated Importance of music (n.s.); **(B)** shows correlation between rated Self Effect and rated impact on Relationship (*r*_*s*_ = 0.33, *p* < 0.01).

A Spearman’s correlation was run to assess the relationship between rated Importance and Self Effect for the whole sample that was not significant, *r*_*s*_ = 0.04, *p* = 0.447; a Spearman’s correlation between Self Effect and assumed change to Relationships yielded a positive correlation, which was statistically significant, *r*_*s*_ = 0.33, *p* = 0.000 (consult Figure 3).

#### Discussion

In Study 2 we found a strong Self Effect for all genres that were above a certain threshold of aesthetic distance. Genres above this threshold exhibited an Aesthetic Self Effect but did not differ significantly from each other. Only the genre pair that was closest on the map elicited a significantly weaker Self Effect compared to the others. The mean score for this transition between the closest genres (Country-to-Folk) was a 4.96, significantly lower (*p* ≤ 0.021) than all other conditions (for a full list of means for Self Effect and rated aesthetic similarity of our items consult Table 4). This result confirmed one of our predictions and led us to conclude that the Aesthetic Self Effects we found might be influenced by the perceived aesthetic dissimilarities of the genres that we used in our vignettes.

We also included a relationship measure in Study 2 that aimed to capture possible social signaling related to the genre preference changes we used in our vignettes. This social change measure indeed correlated within subjects with the reperceived self-measure (*r*_*s*_ = 0.33, *p* = 0.000 see also Figure 3). The impact a preference change in music styles would have on the relationship to friends partially explains why we found an effect. However, the correlation was not as strong as we predicted, hinting at the possible role of other mechanisms driving our effects (consult section “General Discussion” below). For future studies it might be of interest to compare whether such a correlation is stronger for moral domains compared to the aesthetic domain.

For Study 2 we aimed to investigate a wider variety of genre changes. Based on the map from our Pretest we chose genre comparisons to cover certain dimensions on the map (such as movement on the *y*- and *x*-axes, directionality of the change, different distances to the center, etc.) as well as to include movement between several quadrants of the map. The directionality of the genre changes for Study 2 consult the arrows in Figure 2B (additional genre changes used in Study 3 are displayed in Figure 2C; for their selection criteria see the “Materials and Methods” section Study 3).

The relative locations of the genres on our similarity map did not seem to influence whether a change in preference between those genres is perceived as having an impact on a person’s identity. Also, the direction of the change does not seem to matter. We compared the new Pop-to-Classical to the original Classical-to-Pop condition (Study 1) in order to assess whether it makes a difference whether one moves from an outlier on our map toward the midpoint or from the midpoint to the periphery, yet a comparison showed no significant effect, *U* = 674.0, *z* = −0.55, *p* = 0.877, further supporting our finding that mostly aesthetic distance matters. Neither directionality, the central-peripheral dimension, nor movement across specific quadrants on our genre map seems to impact our self-measure.

We then more directly tested the impact of aesthetic distance on our self-measure by comparing the Euclidean distance (i.e., distances in the two-dimensional similarity maps) with the Self Effect 2. As predicted, we found that the map distance was highly correlated to the experience change to identity (*r* = 0.829, *p* = 0.042). The strength of the correlation between participants was somewhat unexpected. Interestingly, the correlation of the directly rated similarities (i.e., only looking at the ratings for the two genres used in the vignette) and the Self Effect did not reach significance (*r* = −0.6, *p* = 0.208). Thus, it is the aesthetic similarity map itself (and not the directly rated distance between two genres) that provides a very good approximation of what figures centrally for the Self Effect. This is a further indication that it is not just the two genres (and possibly the allocated social signaling difference related to those two genres) that influence the assessed effect on identity but possibly other factors as well (including the relative auditory similarity). Further studies, for example including direct auditory stimuli (e.g., sound files), will be needed to determine the full aesthetic dimension of this effect. The results of Study 2 can nonetheless be understood as supporting our hypothesis: the greater the aesthetic distance included in a taste change the greater the impact on the self.

## Study 3: Self-Replication and Extensions to Visual Art

### Introduction

Based on the successful correlation of aesthetic distance and the Self Effect we wondered whether our effect would extend beyond music to other aesthetic domains. Music has been defined as a formal arrangement of sounds that is intimately bound to the emotions (Kivy, 2002; Konecni, 2005). We more generally believe that aesthetic evaluation of art is at its heart emotional (Fingerhut and Prinz, 2018a,b). We therefore chose to also explore visual art. Experiencing art in museum contexts has been related to transformative aesthetic experiences that implicate the self (Pelowski and Akiba, 2011), and artistic practice has long been related to identity, a thread that in 20th century art even became dominant with art exploring transformations of identity, culturally projected (gender) roles, and societal norms related to identity (Meagher, 2002; Mauer, 2005). Among the general population, interest in visual art (or at least “fine art” of the kind exhibited in museums) may be less widespread. We therefore predicted that many participants in a random sample would not self-identity as strongly interested in fine art. In addition, specific patterns of taste in fine art are less firmly associated with subcultures than taste in music. Being a punk rocker or a classical music *aficionado* brings many associations to mind, but a preference for one art genre over another is not necessarily associated with other traits. By exploring whether taste in visual art is associated with identity we can therefore raise the bar for the aesthetic self hypothesis. Does the connection between taste and identity remain even for a domain that is less widely valued and less associated with subcultures?

**FIGURE 4:**
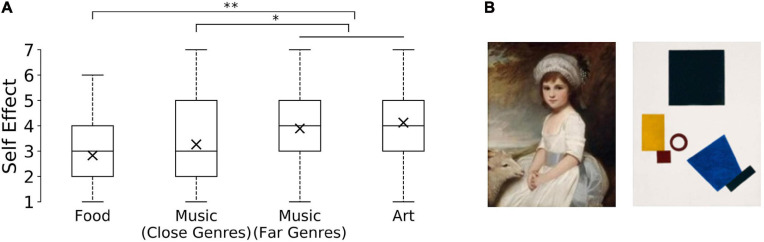
**(A)** Participants’ responses for Self Effect across categories in Study 3, ^∗^*p* < 0.05, two-tailed, ^∗∗^*p* < 0.005, two-tailed. *Y*-axis indicates possible answers on a seven-point Likert scale from 1 (“very much”) to 7 (“not at all the same person”). **(B)** Examples of artworks used as exemplars for preference change in the Art condition in Study 3. Change in preference from left (Romney, 1782) to right image (Malevich, 1915); no titles given in vignette.

In addition to Study 2 we also included aesthetic stimuli instead of only naming the genres or styles. We used a visual representations of two artworks, one traditional-representational and one modern-abstract and asked participants to imagine a change in preference from one artwork to another (for the reproduction of artworks used consult Figure 4B). The rationale behind this was to not just measure the connotations – moral or otherwise – that our wording in descriptive vignettes might have, but the impact on identity based on a more direct aesthetic assessment. This was meant to accompany the measured impact of an aesthetic difference on the self that we attempted with similiarity maps in Study 2. We nonetheless also included a descriptive vignette for the kind of art preference change (Traditional-to-Modern).

### Materials and Methods

237 German adults (*Mdn* age = 30; range 18–71; 42.2% identified as female, 57.0% identified as male) were recruited for the Pretest online via *Clickworker*. Two additional participants were excluded for failing to complete the study. This study was set up as a replication of the Taste-split (between Taste-Aesthetic and Taste-Gustatory) from Study 1 and as an extension to other artforms using the same between-subjects design (10 × 1) with the same wording as before, yielding vignettes with preference changes from Food, Music, and Art categories. Based on Study 1, we chose Food as a possible contrast category to highlight the comparatively strong impact of changes in aesthetic taste, yet also wondered whether the original findings would replicate. Following the “the larger the aesthetic distance, the larger the impact on the self” hypothesis from Study 2 we also aimed to replicate the findings for music. To test whether this would replicate we presented three items with remote distance genre changes (Far-Genres) as well as three close distance genre changes (Close-Genres), interpreting both as independent categories. We therefore decided to compared the genres that were perceived in direct comparison as most distant and those that are least distant in our independent genre comparison study (Pretest 2, consult Supplementary Material B). For the Art category we also included an “exemplar vignette” that presented two artworks that were supposed to indicate the artwork preferred prior to (Figure 4B, left image) and after a change (right image), mirroring the change from more traditional to more abstract, contemporary art from the descriptive vignette. As in Study 1 we first assessed the Self Effect within categories and then compared the Self Effect between categories.

### Results and Discussion

#### Self Effect Within Categories

Using Kruskal Wallis tests we did not find significant differences between Self Effects within the two Music categories: “Far Genres” and “Close Genres” (all *p* ≥ 0.698). Mann–Whitney *U* tests also revealed no significant differences between our items for the Food category and for the two Art conditions (i.e., with or without visualization in the vignette, *p* ≥ 0.98). This led us to retain the following four categories as meta conditions: Art, Music (Far Genres), Music (Close Genres), and Food (for a full list of means per item consult Table 5).

**TABLE 5:** Means and SD for Self Effect in Study 3.

**Category**	**Domain**	**Mean (SD)**	
		**Per domain**	**Per category**
Art	Traditional to abstract (images)	4.58 (1.891)	4.12 (1.837)
	Traditional to abstract (no images)	3.68 (1.667)	
Music (far genres)	Classic to punk	4.00 (1.414)	3.89 (1.733)
	Electronic to gospel	4.00 (1.732)	
	HipHop to classic	3.68 (1.974)	
Music (Close Genres)	Folk to country	3.52 (1.651)	3.26 (1.566)
	Pop to rock	3.20 (1.744)	
	Rock to punk	3.09 (1.213)	
Food	Italian to Asian	2.86 (1.358)	2.83 (1.388)
	Spicy to mild	2.79 (1.414)	

*In descending order of mean per category and then domain.*

#### Self Effect Between Categories

Kruskal Wallis test showed a significant difference between our categories: *H(3)* = 17.478, *p* = 0.001. Subsequent pairwise comparisons using the Mann–Whitney *U* test revealed significant differences for Art (*Mdn* = 4.0) and Close Genres (*Mdn* = 3.0), *U* = 1235.0, *z* = −2.526, *p* = 0.012, and Food (*Mdn* = 3.0), *U* = 668.0, *z* = −3.470, *p* = 0.001, as well as for Far Genres (*Mdn* = 4.0) and Close Genres, *U* = 2001.0, *z* = −2.144, *p* = 0.032 and Far Genres and Food, *U* = 1085.5, *z* = −3.289, *p* = 0.001. No significant difference was found for Close Genres and Food, *U* = 1341.5, *z* = −1.430, *p* = 0.153, as well as for Art and Far Genres *U* = 1658.5, *z* = −0.689, *p* = 0.491 (Figure 4A).

#### Discussion

This study confirmed our key prediction: also changes in visual art preference impact judgments of identity. This corroborated the Aesthetic Self Effect for the domain of visual art, just as we had found it (and replicated) for music, indicating that the relationship between aesthetic taste and identity is not limited to a single artform.

Moreover, as we have already shown with music, the relationship between taste in fine art and our Self Effect does not seem to correlate to the rated Importance of the respective domain. Despite the perceived personal irrelevance of the domain of art in the lives of our participants, they rated an art taste change to have a strong impact on their identity (nominally the domain Art even contained the strongest item for Self Effect, *M* = 4.58). Our importance measure yielded a significant difference across our conditions; KW: *H(3)* = 65.961, *p* < 0.001. *Post hoc* pairwise comparisons using MW tests showed that this was exclusively driven by the Art category (*Mdn* = 4, *M* = 3.61), being significantly less important to our participants than the Music and Food domains (*Mdns* = 6, for all pairwise comparisons *p* < 0.001). This confirmed our assumption that, in the general population, fine art matters less to people than music. This makes the finding with art even more striking, since participants think that taste in fine art relates to identity even though fine art is not especially important to them. This suggests that consciously valuing a certain trait is not required for making that trait central to one’s identity nor that participants personally value that dimension. What seems to matter more given our preliminary data is the domain of the value as well as the distance between the two endpoints of the change in said domain (in our case aesthetic distance).

In this study we did not test whether the degree of aesthetic difference between our stimuli impacts the degree of change in identity. This is because – in contrast to musical genres – we were not confident that people have consistent intuitions about which artistic styles are most similar. Psychological research on the stability of preferences has also demonstrated that human aesthetic stability is also astonishingly low when it comes to visual art stimuli (Pugach et al., 2017). Future work might explore that possibility.

A difference between this experiment and findings reported earlier is that here we failed to find any significant correlations among participant ratings for our Self Effect and Relationship measures within any of our categories. Thus, we did not replicate the pattern for Music in Study 2, which indicated that the degree of change in identity may be associated with impact on membership in social groups. Nor did we find an association between identity change and social relationships for the two conditions in the domain of visual art (Spearman Correlations for all categories: *r* = 0.053–0.177, all *p* ≥ 0.223). Thus, we cannot draw definitive conclusions about the role of social ties and personal relationships in mediating the impact of aesthetic taste change on personal identity. The earlier finding suggests that social relationships may play some role, but they clearly are not the whole story. Taste itself, not mere group membership, may be important to judgments about identity.

## Study 4: the Anaesthetic Self Effect

### Introduction

In the previous studies, most of our vignettes involved a change within a trait or with respect to a domain in which participants had to assume that they already entertain a preference. This is different from the adoption of a what could be considered new trait. For example, we asked participants to imagine a change from one political party to another, or from one taste preference or style to another. One of the exceptions to this pattern were our vignettes about leisure activities: there we presented cases in which somebody picked up an activity they were not interested in before (i.e., beginning to play videogames or taking up hiking).^8^ In Study 4, we were interested in looking at the impact of adopting new traits in the aesthetic domain. We wanted to know if the Aesthetic Self Effect was driven by changes in taste or whether it could also extend to cases where there was a transition from no taste (aesthetic indifference) to aesthetic enthusiasm. We specifically wondered whether changes that would come with picking up an aesthetic interest would lead to a stronger impact on identity compared to picking up a leisure activity. The prediction was that the acquisition of an interest in music, art, or beauty more generally, would have a greater impact on identity than taking up non-aesthetic activities, based on our previous findings. We termed the new conditions “Anaesthetic” conditions as they describe situations in which someone who does not have an aesthetic interest (an “anaesthete”) develops an aesthetic interest or taste in this domain.

We also wanted to explore to what extent dedicating further resources to engagement with the arts (learning an instrument, dedicating your life to a career in music) would elicit a stronger Self Effect compared to merely experiencing them (wanting to listen to music). If the aesthetic self hypothesis is robust, acquisition of interest in the arts should impact identity even if there is no interest becoming a professional. We predicted that dedicating further resources might have an additional effect enhancing the impact on the self, yet that picking up an aesthetic interest in a domain such as music might be sufficient to elicit an Anaesthetic Self Effect.

### Materials and Methods

305 German adults (*Mdn* age = 35; range 18–69, 44.6% identified as female, 54.4% identified as male) were recruited for the Pretest online via *Clickworker*. Nine additional participants were excluded for failing to complete the study.

The set-up and general wording were as in Studies 2–3, producing a 6 × 1 between-subjects design. We asked participants how important the domain (such as music, art, and beauty) was for them and presented them with a change-vignette within this domain in which they had to imagine picking up an activity or interest that they previously did not have. They were then asked to give ratings on our Self Effect and Relationship measures, again anchored on a seven-point Likert scale from 1 (“not at all”) to 7 (“completely”), followed by the same demographic questions as in our earlier studies. We did not replicate the two Leisure conditions from Study 1 but incorporated them as control into our main analysis.^9^

### Results and Discussion

#### Self Effect Within Categories

A Mann–Whitney *U* test revealed no significant difference within our categories. Generally wanting to experience art more (*Mdn* = 5.0) and becoming passionate about visual art (*Mdn* = 5.0) was not significantly different on our Self Effect measure: *U* = 1020.0, *z* = −1.144, *p* = 0.253. Most interestingly, a Kruskal Wallis comparison also revealed no significant difference between our three music conditions *H(2)* = 1.878, *p* = 0.391. For example, “dedicating your life to music” did not render a stronger effect than “wanting to listen to music all the time.” This suggests that the link between aesthetic interest and identity does not depend on vocational choice. This left us with four meta-categories regarding the pick-up of a trait: Leisure, Music, Art, and Beauty. (For a full list of means per item, consult Table 6).

**TABLE 6:** Means and SD for Self Effect of the Anaesthetic conditions in Study 4.

**Category**	**Item** From not caring about beauty (art, visual art, music, hiking, playing video games) to now…	**Mean (SD)**	
		**Per item**	**Per category**
Beauty	…wanting to surround oneself with beautiful objects	4.65 (1.532)	4.65 (1.532)
Art	…wanting to experience art more	4.78 (1.741)	4.60 (1.721)
	…being passionate about seeing visual art	4.42 (1.681)	
Music	…learning to play an instrument and dedicating life to music	4.60 (1.789)	4.40 (1.719)
	…learning an instrument and wanting to play all the time	4.43 (1.631)	
	…wanting to listen to music all the time	4.17 (1.707)	
Leisure	…being passionate about hiking	3.55 (1.955)	3.46 (1.893)
	…being passionate about playing video games	3.37 (1.816)	

*In descending order of means per category.*

#### Self Effect Between Categories

**FIGURE 5:**
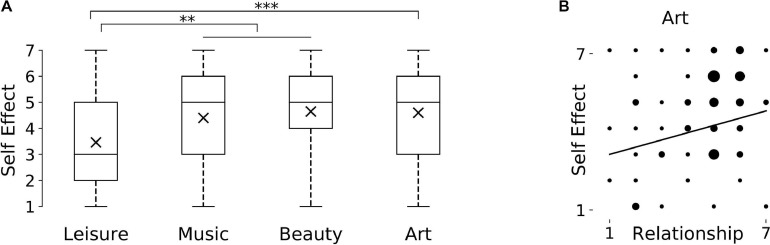
**(A)** Box plots of participants’ responses for Self Effect across categories in Study 4, ^∗∗^*p* < 0.005, two-tailed, ^∗∗∗^*p* < 0.001, two-tailed. *Y*-axis indicates possibble answers on a seven-point Likert scale from 1 (“very much”) to 7 (“not at all the same person”). **(B)** Spearman correlations between Self Effect and relationship measure in Study 4 for art domain (*p* < 0.05); for all other domains no significant correlations were found.

A Kruskal Wallis comparison of the four meta-conditions showed that they were significantly different from each other with respect to our self-measure: *H(3)* = 17.474, *p* = 0.001. Mann–Whitney *U* tests showed that Leisure (*Mdn* = 3.0) was significantly different from all other conditions, i.e., compared to Music (*Mdn* = 5.0) *U* = 3511.5, *z* = −3.401, *p* = 0.001, Beauty (*Mdn* = 5.0) *U* = 1024.5, *z* = −3.368, *p* = 0.001 and Art (*Mdn* = 5.0) *U* = 2003.0, *z* = −3.72, *p* = 0.000 (Figure 5A). The three Anaesthetic conditions were not significantly different from each other (*p* ≥ 0.356).

Using a Kruskal Wallis Comparison for the Importance scores revealed a significant difference between our meta-conditions in this measure: *H(3)* = 63.324, *p* < 0.001). This effect was driven by the Art condition (*Mdn* = *4.0*, *M* = 4.0): Art was significantly less important to our participants than all other domains (Leisure *Mdn* = 6.0, *M* = 5.75, Music *Mdn* = 6.0, *M* = 5.66, Beauty *Mdn* = 6.0, *M* = 5.75, *p* = 0.001).

As before, a Spearman’s correlation used to assess the relationship between ratings of Importance and Self Effect for each category was not significant (*p* ≥ 0.144). A Spearman’s correlation between Self Effect and assumed change to Relationships ratings yielded a positive statistically significant correlation only for the Art condition, *r*_*s*_ = 0.278, *p* = 0.006 (Figure 5B). All other correlations were not significant (*p* ≥ 0.355).

#### Discussion

Our main question in Study 4 was whether the acquisition of an interest in the arts would have an impact on identity, as we have previously shown for changes in aesthetic taste. Our results provide an affirmative answer. That is, the acquisition of an aesthetic interest or of an engagement with the arts has a large impact on identity, and decidedly so compared to the acquisition of leisure activities. This was somewhat surprising, given the already strong impact of leisure changes in Study 1. It provides further support to our hypothesis that aesthetic values are among the more impactful contributors to identity. An even more surprising finding was that we found no difference between a pure aesthetic condition (coming to care about an aesthetic domain such as music) and conditions that additionally included an activity or commitment (learning to play an instrument and dedicating life to music). This gives support to an inherently aesthetic effect independent of associated dedication of resources or activities. It is the presence of aesthetic engagement or experience itself, not the associated activities, that seems to drive judgments about identity here.

Another general question one might ask is whether the acquisition of a new domain of interest would be perceived as having a larger impact on the self, compared to a preference change *within* a domain. We addressed this by checking whether Aesthetic conditions (the change of preference within an aesthetic domain) and Anaesthetic conditions (the picking up an aesthetic domain) would yield a difference with respect to our self-measure. First of all, Kruskal Wallis tests showed no significant difference *within* our Taste Aesthetic conditions^10^ and *within* our Anaesthetic conditions: Taste Aesthetic: *H(9)* = 6.855, *p* = 0.739, Anaesthetic *H(4)* = 4.401, *p* = 0.493. We then combined all Aesthetic and all Anaesthetic conditions into two groups. The comparison between those grouped variables showed a significant difference: Aesthetic conditions (*Mdn* = *4*) showed a significantly weaker Self Effect compared to Anaesthetic Conditions (*Mdn* = *5*), *U* = 79001.5, *z* = 4.426, *p* < 0.001.

In Study 4 we also included changes in the general interest in beauty and the arts that differed from the other conditions in that they did not specify the art form or cultural artifacts, such as music, visual art, paintings with respect to which the engagement changes. Beauty elicited the second largest Self Effect for a single vignette (*M* = 4.65) only exceeded by the general interest in art (*M* = 4.78). This might be due to the generality of the proposed change or due to the kind of valuing related to the conditions: changes in art engagements and beauty engagements might be of central importance to our selves. That changes in beauty preferences are important might additionally supported by the finding in Study 3 were the perceived beauty of the two exemplar images (consult Figure 4B) was also very different.^11^ The beauty condition elicited the third largest Self Effect (*Mn* = 4.58). Future studies will have to investigate how these findings relate to the overall concepts of art and beauty.

## General Discussion

### Did We Test Identity?

Throughout this paper, we have focused on the relationship between aesthetic taste and judgments of personal identity. One might wonder whether a study of intuitions can shed some light on the nature of identity itself, rather than merely revealing how we make such judgments. We think the answer is yes, because identity is not a deep metaphysical fact, but rather a construction. That is, it is at least partially up to us to choose which of our many traits matter for retaining the same identity. When we refer to someone as the same person over various changes, we are not necessarily referring to numerical identity (Starmans and Bloom, 2018), but rather to the qualitative features that matter to us when keeping track of people. Qualitative identity is what we care about when we wonder whether a loved one has changed or whether a convicted criminal is a new person after undergoing a moral change while incarcerated (Gomez-Lavin and Prinz, 2019). It is this notion of identity that arises when we express existential concerns that a dramatic experience in life could change who we are (see Schechtman on identity crises, 1996: 74).

It may be surprising to find an empirical link between aesthetics and identity, since there has been so little exploration of this connection in either empirical aesthetics or psychology. But, again, we think the connection does resonate with common sense. We often hold on to our youthful tastes in music and art, and we make these tastes priorities in our free time. We also talk about art that “speaks to me” or “expresses who I am.” In a recent phenomenological interview study conducted in a contemporary art museum we found that participants chose artworks as their favorites that fulfill such personal criteria over other artworks they found interesting but that did not “reflect who I am!” (Fingerhut et al., n.d..). Such phrases indicate that our qualitative sense of identity is bound up with our personal art preferences, along with other traits. Clearly the arts matter a great deal to us, and the present findings suggest that this importance goes beyond mere liking and is bound up with our sense of who we are. Changes in the general interest in the arts might also indicate a change in a general outlook including an openness to new experiences and cognitively demanding situations, which have been related to an artistic personality (Chamorro-Premuzic et al., 2007). This outlook might also forge social bonds with groups or people that hold the same aesthetic values we endorse. Both genre preferences (e.g., being an opera buff) and an overall interest in the arts (e.g., being an “culture maven”) might be thought of as human types. We hope that the findings here, though preliminary, will help prompt further investigation into the aesthetic self.

### Relation to Other Findings

Recent empirical work has found a consistent and robust association between judgments about personal identity and moral traits, values, and behaviors. The so-called “moral self” was established by showing that changes in an individual’s set of moral values elicited larger impacts on perceived identity than other candidates stemming from the philosophy of identity, particularly the notion that continuity of autobiographical memory should matter most (Strohminger and Nichols, 2014; Prinz and Nichols, 2016; Heiphetz et al., 2017). A few prior studies examined aesthetic traits and preference; however, these traits were found to be less central to identity compared to moral ones, prompting the authors to suggest that they found “strong and unequivocal support for the essential moral self hypothesis” (Strohminger and Nichols, 2014: 168).

Our studies showed that aesthetic traits can play a similar role. Why did we find an Aesthetic Self Effect that those studies did not find? First, we used a different design. In our between-subject design we presented each participant with only a single change in their life that they had to rate on its possible impact on their identity. The *rationale* for this was that we wanted participants to imagine more vividly such a change (with the experiential components coming with it). The same *rationale* also led us to include a question regarding the domain itself (“How important is x for you?”) in order to prime thinking about a domain before presenting the change vignette. Most other studies implemented a within-subjects design with several vignettes presented simultaneously which allowed participants to directly compare moral changes to changes in other categories, including in aesthetic traits. Any effect found in such studies might be confounded by overt comparisons between aesthetic and moral domains (the former perceived as more leisure related, e.g., music, the other as more serious, e.g., politics and religion).

Another explanation for the divergence in findings could be that Strohminger and Nichols (2014) presented participants with loss of traits whereas we presented participants with the possibility of *gaining* a trait—by, for example, going from disinterestedness about music to imagining that they would actively engaging with it. Perhaps participants more severely judge losing a moral trait than to lose an aesthetic interest. In order to assess this possibility, we conducted a follow-up study. 236 German adults (*Mdn* age = 31, range 18–68; 46.2% identified as female, 53.0% identified as male) were recruited online via *Clickworker* (2 additional participants were excluded for failing to complete the study). We reversed several of our conditions in which we described the gaining of a trait: the two conditions from our leisure category (Study 1) and one item of each of our anaesthetic categories (Study 4): music, art, and beauty. Pairwise comparisons of reverse to original conditions using Mann–Whitney *U* tests yielded no significant difference between gaining or losing a trait for any of those conditions (*p* ≥ 0.159). The reverse condition interestingly attenuated our effect though, with all measure moving more toward the mid-point of our scale with only the “losing your interest in beautiful things” condition (*Mdn* = 5.0, *M* = *4.69)* remaining significantly different from the leisure category yet the trend fully intact (*Mdn* = 4.0, *M* = *3.94)*, *U* = 737.0, *z* = −2.070, *p* = 0.038, for full list consult Supplementary Material C).

### Methodological Considerations: Imagining Preferences and Changes

Our experimental set-up (as well as the experimental literature on personal identity we build upon) focuses on counterfactual changes that participants have to imagine based on descriptive vignettes presented to them. They then have to rate the perceived change to identity that this might entail. Such a methodological set-up has advantages but also serious limitations. Any results we might find could, for example, be confounded by the vividness in which subjects are able to imagine a trait, preference, or activity described in our vignettes. We asked subjects to imagine having a trait (e.g., liking classical music) and then to undergo a change with respect to this trait (e.g., to then like pop music). Whether a participant is indeed a classical music lover might affect the perceived change to identity: they might report a higher Self Effect given that they experiences more vividly what such a preference entails (having, e.g., more associations and an affective bond related to it) and therefore also rate the moving away from it as a bigger change to the person they are.^12^ Conversely, it may also be the case that asking participants to imaging changing with respect to an unfamiliar or “alien” trait might elicit a stronger Self Effect (e.g., imagining “dedicating your life to music” for a music grouch). We did not account for such factors in our studies. Future studies might provide this information by asking participants whether they actually entertain a preference with respect to an aesthetic genre or style that is presented in the vignette. In our studies we aimed for something similar, yet in a more general fashion: we asked how important the respective domain (i.e., Politics, Religion, Music, Art, Beauty, etc.) employed in the vignette was for the participants. This was done prior to asking them to imagine a counterfactual change within this domain and partly to prepare them for the change presented in the following vignette (i.e., to enhance their vividness). Interestingly, we did not find an effect of rated self-importance of a domain on our self-measure in any of our studies (Spearman’s correlation used to assess *within* subject relations between rated Importance and Self Effect per each category were not significant; *p* ≥ 0.144).

It was not our goal to assess how enduring or convertible aesthetic taste is over the lifespan, nor whether our participants actually underwent taste or personality changes in their lives. Other studies have explored aesthetic preference stability and malleability across different time spans, by focusing, for example, on the stability of preferences for geometric figures (McManus et al., 2010), the impact of sensorimotor experience on affective relation to dance (Kirsch and Cross, 2018), or the relative instability for aesthetic ratings for visual art (Pugach et al., 2017) as well as the stability and strength of preferences for music (Schäfer, 2016). There are also more general models of how sensory inputs, motivations, and reward might influence aesthetic preferences over a life span (Aleem et al., 2020). We are currently conducting follow-up studies exploring more directly the occurrence of significant taste changes by letting participants self-report experienced taste changes in their lives followed by an assessment of whether they judge this to have been a change in their identity. Generally, we believe that the aesthetic-identity link constitutes an exciting new field of research to which we hope to have contributed with our present studies.

### Mechanisms and Motives: Emotions and Social Signaling

In our studies we did little to directly investigate why aesthetic values are linked to identity. What mechanisms mediate this link? Why might the association to the arts matter to people? Regarding mechanisms, social signaling and bonding might play a role: both morals and aesthetics have social significance. Tracking a person’s moral identity might help us to determine whether somebody might be helpful or harmful toward oneself (Goodwin, 2015). Aesthetics might be central in the very same respect, helping us to identify whether somebody shares a general outlook with ourselves. Music and art taste can indicate moral values, cultural identity, and social class, and they also are a source of bonding in their own right. Someone with similar taste may be more likely to treat you preferentially (Tajfel, 1970). Future work can explore this possibility: we might perceive a change in taste as a blow to our identity because it indicates that we might belong to a different social group given such a taste. We believe that this view of the self as grounded in the relation to others holds a great plausibility. We also aimed to test this possibility by including a social change measure: we asked participants to what extent they think whether the change presented would impact their relationship to their friends. The findings here were inconclusive: We did find a correlation for some conditions but not for all. Assessing this correlation across all studies we find a positive correlation between Self Effect of our aesthetic conditions and change in relationship among friends (using Spearman: *r* = 0.263, *p* < 0.001). This effect was more pronounced than the one we found for all our Anaesthetic conditions (*r* = 0.148, *p* = 0.01), implying that to some extent the impact of taste changes (i.e., aesthetic preference changes) might be grounded in the change in social relationship they indicate.

Emotions and social factors may work together. Emotionally expressing a preference signals one’s membership in likeminded social groups in a fast and direct way. Research might explore the extent to which a display of preference both impacts others and increases a sense of affiliation. One might then explore whether such shared dimensions of taste lead to strong links between taste and identity. Emotional signaling with respect to taste may simultaneously increase social identity (preferential affiliation) and the aesthetic dimensions of personal identity (the degree to which the shared dimension of taste would impact identity were it to change). The link to emotions might also be established in a more direct way as well: aesthetic evaluations and our appreciation of the arts may be affective by nature (Prinz, 2011). Aesthetic attitudes can be construed as motivational states that dispose our embodied and situated selves to engage with cultural artifacts (Fingerhut and Prinz, 2018b, 2020). Behavioral studies point to links between emotional and social processes in domains of taste: the less access participants have to their emotions and the emotions of others (emotional contagion) the less interesting the artworks and the lower liking ratings they give (Gerger et al., 2018). Certain emotional profiles also correlate with the liking of specific art genres. it has, for example, been shown that participants with higher scores for neuroticism prefer abstract art over more classical representational artworks (Furnham and Walker, 2001; Lyssenko et al., 2016). Again, more studies are conducted on this in the moral domain compared to aesthetics and the arts, yet future studies might explore this joint link to the emotions and its contribution to a sense of self.

### Summary and Outlook

We have explored how the relationship between aesthetic preferences and personal identity is conceived within a general population. We sought to test the hypothesis that people would regard changes in aesthetic taste as changes in the self. This hypothesis emerged as an extension of recent research on the relationship between moral values and judgments of identity, but it also falls in line with previous work in psychology and sociology. There has been prior psychological work correlating aesthetic preferences and personality, and sociologists have long associated aesthetic preferences with aspects of social identity such as status, class, and membership in subcultures. In addition, ordinary language includes phrases such as “art person” and “music person.” Lastly, work on aesthetic evaluation in neuroscience, psychology, and philosophy attests to the centrality of emotional processing for aesthetics and art appreciation (Brown et al., 2011; Fingerhut and Prinz, 2018a, 2020; Menninghaus et al., 2019) revealing a similarity to moral judgments. None of these observations directly speaks to our hypothesis, but they help to motivate the investigation that we undertook here. To our knowledge this is the first systematic exploration of the hypothesis that changes in taste and aesthetic values would be perceived as impacting the identity of the person undergoing the change.

Our experiments established a link between identity and taste. We interpret this as showing that people have “aesthetic selves” – that is, we attribute to ourselves and others a dimension of identity that revolves around preferences for art. This includes a general interest in the arts (being an art person or music person) as well as the specific artistic taste one has. A shift in taste is judged to be a shift in identity. This transformational capacity through our engagements and preferences in the arts is the Aesthetic Self Effect. Future work might also investigate whether the effect extends to interests and taste in architecture, film, theater, dance, and the other arts. One particular interesting domain of taste in this respect is fashion, which in a unique way already combines the arts and the expression of identity.

It was not a primary goal here to determine the mechanisms underlying this effect. The impact on social group membership was explored in a preliminary way with inconclusive results. Another possibility suggested at the outset is that aesthetic values have a basis in emotions and that emotional continuity is perceived as important to identity. Alternatively, the relationship could be mediated by our participants’ assumptions about artistic taste and moral values, cultural affiliations, class, and other variables that we did not measure here. Future work should investigate these and other possibilities.

Research on aesthetic psychology has intensively studied preference and aesthetic experiences, but, outside of sociology, these preferences are rarely linked to identity. This opens up new lines of investigation. Perhaps liking art is not just a matter of finding intrinsic features of artworks appealing, but also involves asserting something about one’s self. The findings here are the first of their kind, and they have implications for both the study of personal identity and the study of aesthetic psychology. Each domain of inquiry has tended to ignore the other. If aesthetics is a component of identity the menu of leading philosophical theories needs to be expanded. We tend to think of taste and the arts as merely recreational. Taste also is often seen external to the individual in so far as it is culturally transmitted and involves relationships to cultural artifacts. We have shown that taste is perceived as being central to who we are. Therefore, theories of identity should be updated to recognize that personal identity may be aesthetic, cultural and situated, in that it involves relations to social and material things outside the individual organism.

## Data Availability Statement

The raw data supporting the conclusions of this article will be made available by the authors, without undue reservation.

## Ethics Statement

The studies involving human participants were reviewed and approved by CUNY University Committee on Activities Involving Human Subjects. The patients/participants provided their written informed consent to participate in this study.

## Author Contributions

JF, JG-L, and JP conceptualized the experiments. JF, JG-L, CW, and JP reviewed and edited the manuscript. JF, JG-L, and CW acquired and curated the data, analyzed the data, and created the tables. CW created the figures. JF and JP created the first draft of the manuscript. JF supervised the project. All authors contributed to the article and approved the submitted version.

## Conflict of Interest

The authors declare that the research was conducted in the absence of any commercial or financial relationships that could be construed as a potential conflict of interest.

## Footnotes

1 All studies reported in this paper were conducted in German. Translations into English are from the authors *JF* and *CW* (consult Supplementary Material A1).

2 The non-normality finding was robust and consistent throughout all the datasets reported in this paper. Given the distributions of our data, alongside our use of ordinal measures, we therefore will report the results of non-parametric tests throughout this paper. Consult Diaz et al. (2017) and Gomez-Lavin and Rachar’s (2019) recent work in experimental philosophy which both serve to highlight the use of non-parametric methods for similar vignette styled experiments.

3 The slightly lower ratings for political affiliation change compared to religion (both moral conditions) might have been an artifact of the rather leveled political landscape in Germany in 2017 during the time we conducted Study 1. Especially the two main parties named in our vignettes, CDU (mid-conservative) and SPD (mid-left-wing) were in a coalition at the time and, might not have been perceived as being that different in political-moral outlook. (The conservative party, e.g., at the time promoted pro-choice and pro-immigration views following Angela Merkel’s “We can do it!” proclamation regarding increased refugee intake in Germany). We would predict a stronger change in identity with similar vignettes for countries with a two-party system, such as the United States, where switching from supporting the Republican Party to supporting the Democrat Party might be perceived as a much bigger change in identity. This points toward a need for cross-cultural studies to examine possible differences for what participants of different cultural backgrounds consider being central for their perceived identity. We aimed to mitigate possible cultural differences and other confounds by including a direct measure (Q1 and Q2) of perceived moral (and taste) change in our Pretest that indeed showed a moral effect for our Politics condition.

4 The latter has a longstanding history in the philosophy of aesthetics and has been prominently discussed in Hume’s treatment of the development of taste of the “ideal judge” regarding artistic works and his claims that taste is the basis for both, moral and aesthetic judgments (Hume, 2000, [1739]).

5 Classical music may, e.g., be associated with a kind of German national identity and the Self Effect therefore not driven by aesthetics in this case. We tested for this possibility by asking a subset of our participants in Study 2 (*n* = 53) whether “Classical music is a particularly German art form,” to which our participants largely agreed giving a median response of 5 (*M* = 4.53, SD = 1.512) on a scale of 1 to 7, with seven being “very much.” This is compared to lower responses for Pop as a German art form (*Mdn* = 4, *M* = 3.53, SD = 1.207). A Wilcoxon signed-rank test showed that classical music was considered significantly more German (*Z* = 20, *p* < 0.001). This vindicated our decision to consider other genres beyond classical music.

6 Here the study was exploratory: it might be that one of the dimensions of our map actually covered a relation of genres to the effect that it would change a person more significantly if they switched their preferences between genres aligning on this dimension.

7 Per Tucker’s coefficient of congruence.

8 Another example of this was our Religion condition, where we asked participant to imagine a change from being a faithful believer to becoming an atheist, which, under some descriptions could also be considered a loss of a trait. See also the “General Discussion” for this.

9 A side effect of this was that we did not have the additional relationship measure for leisure that we only introduced after Study 1.

10 To this end we compared all Aesthetic conditions from studies 1–3. We excluded the close-genre conditions for Music that we considered to not elicit an Aesthetic Self Effect, due to the aesthetic similarity of the genres.

11 This was assessed in a different study with an independent sample of participants. The Romney (left image) received average ratings of 77 out of 100 for beauty (being among the highest rated), while ratings for the Malevich were below the midpoint of our scale. The exemplar vignette in the present study therefore possibly indicates a change in preference for beauty by switching between the two.

12 Thanks to Reviewer 2 for pointing this out.
