# Supplementary material for: The Aesthetic Self. The Importance of Aesthetic Taste in Music and Art for Our Perceived Identity
Source: Front Psychol. 2021 Mar 9;11:577703. doi: 10.3389/fpsyg.2020.577703 (PMC7985158; doi:10.3389/fpsyg.2020.577703)

# A1: Fingerhut, Gomez-Lavin, Winklmayr, Prinz: The Aesthetic Self

SUPPLEMENTARY MATERIAL A1 | List of instructions presented to the participants after “Informed Consent”. Column 1 lists the question category and the studies (S) and pretests (P) where each category was used. We included one examples of a question (in the original German as well as an English translation, *in italics*) are presented in the second column. The possible responses are listed in column 3. We also provide screenshots of two example questions as they were presented to the participants.

| **Category** | **Wording** | **Answer options** |
| --- | --- | --- |
| **Introduction** | Wir bitten dich genau über Veränderungen nachzudenken, die du erfahren könntest. Einige Veränderungen im Leben – wie zum Beispiel ein neuer Haarschnitt – haben wahrscheinlich eher wenig Einfluss darauf, wer du bist. Andere hingegen, können einen enormen Einfluss haben. Für eine bestimmte Veränderung werden wir dich bitten, dich selbst zu fragen, “Wäre ich die gleiche Person?“, wenn diese Veränderung passiert. Wie würde ich mich selbst nach dieser Veränderung sehen?    *We ask you to think carefully about changes you might experience. Some changes in life - such as a new haircut - probably have little effect on who you are. Others, however, can have a big impact. For the presented example of a change, we want you to ask yourself: ‘Would I be the same person?’ if this change takes place. How would I regard myself after the change?* | none |
| **Importance** | Wie wichtig ist dir Musik? | gar nicht wichtig (1) – sehr wichtig (7) |
| P1, S1, S2, S3, S4 | *How Important is Music to you?* | *not at all important (1) – very important (7)* |
| **Self Effect** | Nimm an, dein Musikgeschmack hat sich drastisch verändert. Stell dir zum Beispiel vor, dass du nur klassische Musik magst und dann ausschließlich Popmusik hören willst. Würdest du dich als die gleiche Person betrachten? | völlig dieselbe Person (1) –  gar nicht dieselbe Person (7) |
| S1, S2, S3, S4 | *Assume your taste in music has changed drastically. Imagine, for example, that you used to only like classical music but have now grown to only like pop music. Would you regard yourself as the same person?* | *very much the same person (1) – not at all the same person (7)* |
| **Relationship** | Wie sehr denkst du, dass sich das Verhältnis zwischen Freunden oder Freundinnen verändert, wenn einer von beiden seinen Geschmack so drastisch verändert? | gar nicht (1) – sehr (7) |
| S2, S3, S4 | *How much do you think the relationship between friends will change, if one of them so drastically alters their taste?* | *not at all (1) – very much(7)* |
| **German Artform** | Wie sehr denkst du, dass klassische Musik eine deutsche Kunstform ist? | gar nicht (1) – sehr (7) |
| S4 | *How much do you think Classical Music is a German art form?* |  |
| **Moral and Aesthetic Change** | - Wie sehr denkst du, dass diese Veränderung eine Veränderung in deinem Geschmack widerspiegelt? - Wie sehr denkst du dass diese Veränderung eine Veränderung in deinen moralischen Werten widerspiegelt? - Wie sehr denkst du, dass du nach der Veränderung ein anderer Typ von Mensch bist? | Gar nicht (1) – sehr (7) |
| P1 | - *How much do you think that this change represents a change in taste?* - *How much do you think that this change represents a change in your moral values?* - *How much do you think you will be a different person after this change?* | *not at all (1) – very much (7)* |
| **Genre Change** | Wie ähnlich sind diese Musikstile? | *Gar nicht (1) – völlig (100)* |
| P2 | *How similar are these Genres?* | *Not at all (1) – completely (100)* |

Screenshots of Likert scale and similarity rating used in Qualtrics


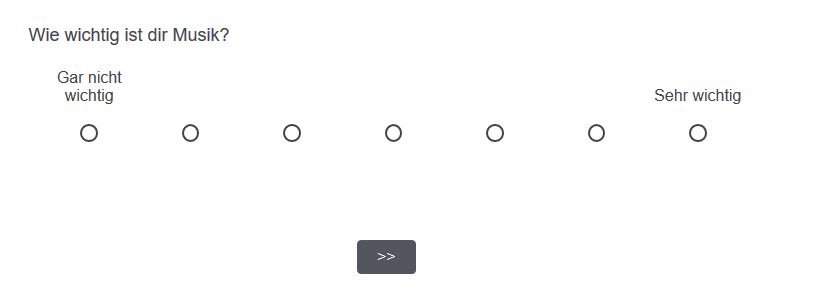

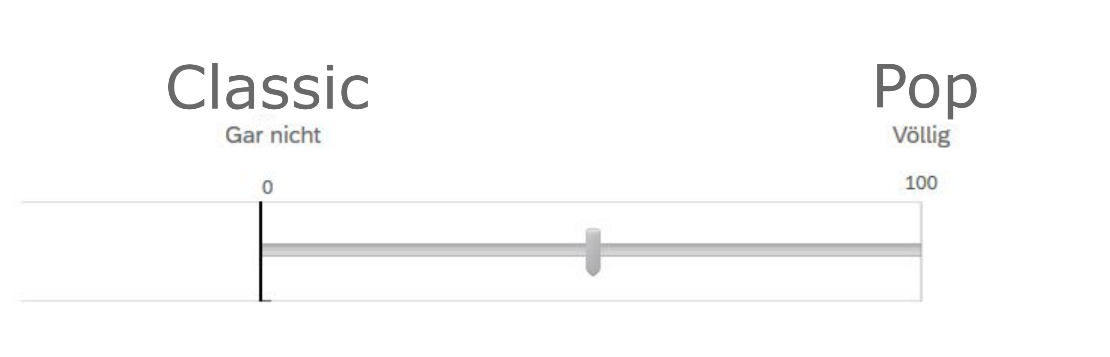

Supplement: Supplementary file 1 [file Table_1.DOCX]
