# Supplementary material for: The Aesthetic Self. The Importance of Aesthetic Taste in Music and Art for Our Perceived Identity
Source: Front Psychol. 2021 Mar 9;11:577703. doi: 10.3389/fpsyg.2020.577703 (PMC7985158; doi:10.3389/fpsyg.2020.577703)
Supplement: Supplementary file 2 [file Table_2.DOCX]

# A2: Fingerhut, Gomez-Lavin, Winklmayr, Prinz: The Aesthetic Self

SUPPLEMENTARY MATERIAL A2 | List of instructions, demographics and personality measures used in Studies 1-4 and Pretests. Questions are grouped by category and we list the original German wording as well as an English translation (in *italics*). The column on the right indicates in which study (S) or pretest (P) the respective item was used.

| **Category** | **Wording** | **Answer Options** | **Studies** |
| --- | --- | --- | --- |
| **Gender** | Sag uns bitte dein Geschlecht /  Wie würdest du dein Geschlecht beschreiben? | männlich; weiblich; keine der beiden Optionen/  männlich; weiblich; keine der beiden Optionen; keine Antwort | S1  P1, P2, S2, S3, S4 |
|  | *Please tell us your gender/*  *How would your describe your gender* | *male; female; none of the above/*  *male; female; none of the above; prefer not to answer* |  |
| **Age** | Bitte wähle deine Altersspanne /  Wie alt bist du? | 18-24;25-35;36-50;>50 /  Freier Eintrag | S1  P1, P2, S2, S3, S4 |
|  | *Please choose your age box/*  *How old are you* | *18-24;25-35;36-50;>50 /*  *textbox entry* |  |
| **Area of**  **Living** | Wie würdest du die Gegend beschreiben, in der du lebst? | Städtisch; vorstädtisch; ländlich | P1, P2, S2, S3, S4 |
|  | *How would you describe the area you live in?* | *urban; suburban, rural* |  |
| **Religion** | Wie religiös bist du? | Gar nicht (0) – sehr (7)/  Gar nicht (0) – sehr (10) | S1  P1, P2, S2, S3, S4 |
|  | *How religious are you* | *Not at all (0) – very much (7)/*  *Not at all (0) – very much (10)* |  |
| **Politics** | Wie würdest du deine politischen Werte beschreiben? | Sehr konservativ (0) – sehr links (7)/  Sehr konservativ (0) – sehr links (10) | S1  P1, P2, S2, S3, S4 |
|  | *How would you describe your political values?* | *Very conservative(0) – very left-wing (7)*  *Very conservative(0) – very left-wing (10)* |  |
| **Party**  **Identification** | Mit welcher Partei identifizierst du dich am meisten? | CDU; SPD; die Linke; die Grünen; FDP; AfD; Piraten;NPD; Sonstige | S1, P1,P2, S2, S3, S4 |
|  | *With which party do you identify the most?* | *CDU; SPD; die Linke; die Grünen; FDP; AfD; Piraten; NPD; others* |  |
| **Party voted** | Welche Partei hast du bei der letzten Bundestagswahl gewählt? | CDU; SPD; die Linke; die Grünen; FDP; AfD; Piraten; NPD; Sonstige | S2, S3, S4 |
|  | *Which party did you vote for in the last election?* | *CDU; SPD; die Linke; die Grünen; FDP; AfD; Piraten; NPD; others* |  |
| **Ethnicity** | Wie würdest du deine ungefähre ethnische Zugehörigkeit oder deinen familiären Hintergrund beschreiben? | Osteuropäisch; südländisch, nordisch; deutschstämmig; asiatisch; afrikanisch; mittlerer Osten; türkischstämmig; nordamerikanisch; latino, ich will keine Angaben machen; andere | P1, P2, S2, S3, S4 |
|  | *How would you describe your Ethnicity/family background?* | *Eastern-european; Southern-european, Nordic; of German Descent; Asian; African; Middle East; of Turkish descent; Northern American; Latino; no Answer/others* |  |
| **Museum**  **Visits** | Wie oft besuchst du Kunstmuseen und/oder Galerien? | Nie; etwa einmal im Jahr; mehrmals im Jahr; monatlich; wöchentlich | P1, P2, S2, S3, S4 |
|  | *How often do you visit Art Museums/galleries?* | *Never; approximately once a year; several times a year; monthly; weekly* |  |
| **Museum Education** | Hast du eine Form von formaler Ausbildung erhalten, entweder als Künstler oder in Kunstgeschichte? | Keine; bis zu ein Jahr; bis zu zwei Jahr; mehr als zwei Jahre; mit einem Abschluss | P1, P2, S2, S3, S4 |
|  | *Did you receive any formal education as an artist or art historian?* | *None; up to one year; up to two years; more than two years:; with a degree* |  |
| **Art Liking** | Wie sehr magst du Kunst? | Gar nicht (0) – sehr (10) | P1, P2, S2, S3, S4 |
|  | *How much do you like Art?* | *Not at all (0) – very much(10)* |  |
| **Art preference** | Welche Art von Kunst magst du lieber? | realistische Bilder und klassische Kunst; abstrakte und konzeptionelle Kunst | P1, S2, S3, S4 |
|  | *Which type of Art do you prefer?* | *Realistic pictures and classical art; abstract and conceptual art)* |  |
| **Education** | Was ist dein höchster Schulabschluss? | Haupt- oder Realschule; Abitur; Bachelor; Master,Magister,Diplom; Doktor (phil o.a.); Sonstige; Kein Schulabschluss | P1, P2, S2, S3, S4 |
|  | *What is your highest degree of education* | *High school, Bachelor, Master, PhD, other, none* |  |
| **Germanness** | Wie sehr identifizierst du dich damit,  Deutscher zu sein? | Gar nicht (0) – sehr (10) | P1, S2, S3, S4 |
|  | *How much do you identify with being German?* | *Not at all (0) – very much(10)* |  |

# **Ten Item Personality Measure (TIPI)**

The TIPI consists of ten questions where participants are asked to what degree they would assign themselves a certain personality trait. Each questions starts with the phrase “I see myself as…” followed by two adjectives describing a personality aspect. The participant can then select their answer from a 7-point scale. The Tipi was used in Pretest 2 as well as Studies 2 and 4 (for future cross-cultural comparisons) In the following we list the wording in the original German as well as an English translation

**Ich sehe mich selbst als… *I see myself as….***

| Extravertiert, begeistert |  | *extroverted, enthusiastic* |
| --- | --- | --- |
| Kritisch, streitsüchtig |  | *critical, argumentative* |
| Zuverlässig, selbstdiszipliniert |  | *reliable, self-disciplined* |
| Ängstlich, leicht aus der Fassung zu bringen |  | *anxious, easy to lose poise* |
| Offen für neue Erfahrungen, vielschichtig |  | *open to new experiences, complex* |
| Zurückhaltend, still |  | *guarded, quiet* |
| Verständnisvoll, warmherzig |  | *understanding, warm-hearted* |
| Unorganisiert, achtlos |  | *unorganized, careless* |
| Gelassen, emotional stabil. |  | *Calm, emotionally stable* |
| Konventionell, unkreativ. |  | *Conventional, uncreative* |
|  |  |  |

| **Antwortmöglichkeiten***:* | ***Answers:*** |
| --- | --- |
| Trifft überhaupt nicht zu, Trifft größtenteils nicht zu, Trifft eher nicht zu, Trifft eher nicht zu, Weder zutreffend noch unzutreffend, Trifft eher zu, Trifft größtenteils zu, Trifft voll und ganz zu. | Disagree strongly, Disagree moderately, Disagree a little, Neither agree nor disagree, Agree a little, Agree moderately, Agree strongly. |
