# Supplementary material for: The Aesthetic Self. The Importance of Aesthetic Taste in Music and Art for Our Perceived Identity
Source: Front Psychol. 2021 Mar 9;11:577703. doi: 10.3389/fpsyg.2020.577703 (PMC7985158; doi:10.3389/fpsyg.2020.577703)
Supplement: Supplementary file 3 [file Table_3.DOCX]

# B: Fingerhut, Gomez-Lavin, Winklmayr, Prinz: The Aesthetic Self

SUPPLEMENTARY MATERIAL B | Table with list of distances between genres in Euclidean distance on the Map and “Rated Similarity” from Pretest 2. In descending order of “Map Distance”

| **Genres** | **Map** | **Rated** |
| --- | --- | --- |
|  | **Distance** | **Similarity** |
|  |  |  |
| Classical vs. HipHop/Rap | 1.5438 | 10.067 |
| Gospel vs. Punk | 1.4665 | 11.711 |
| Electronic vs. Country | 1.4161 | 10.978 |
| Electronic vs. Folk | 1.3853 | 11.422 |
| Gospel vs. HipHop/Rap | 1.3393 | 18.311 |
| Folk vs. Punk | 1.3387 | 13.867 |
| Classical vs. Punk | 1.3321 | 06.111 |
| Electronic vs. Gospel | 1.3112 | 10.556 |
| Classical vs. Country | 1.3038 | 19.622 |
| Jazz vs. HipHop/Rap | 1.2297 | 22.289 |
| Country vs. Pop | 1.2292 | 37.089 |
| Jazz vs. Punk | 1.1516 | 14.511 |
| Electronic vs. HipHop/Rap | 1.1480 | 44.244 |
| Classical vs. Pop | 1.0971 | 19.378 |
| Classical vs. Folk | 1.0886 | 26.778 |
| Folk vs. HipHop/Rap | 1.0292 | 15.222 |
| Gospel vs. Rock | 0.9955 | 23.933 |
| Folk vs. Rock | 0.9474 | 32.133 |
| Country vs. Rock | 0.9322 | 33.889 |
| Jazz vs. Country | 0.9272 | 33.311 |
| Gospel vs. Pop | 0.9021 | 32.089 |
| Electronic vs. Pop | 0.8996 | 38.133 |

| **Genres** | **Map** | **Rated** |
| --- | --- | --- |
|  | **Distance** | **Similarity** |
|  |  |  |
| Classical vs. Rock | 0.8824 | 23.511 |
| Electronic vs. Classical | 0.8752 | 14.067 |
| Jazz vs. Electronic | 0.8700 | 19.000 |
| Country vs. HipHop/Rap | 0.7960 | 11.600 |
| Jazz vs. Pop | 0.7680 | 31.067 |
| Classical vs. Gospel | 0.7482 | 37.956 |
| Country vs. Gospel | 0.7453 | 37.156 |
| Jazz vs. Folk | 0.7249 | 31.378 |
| Rock vs. HipHop/Rap | 0.7248 | 27.356 |
| Folk vs. Pop | 0.6792 | 42.178 |
| Pop vs. Punk | 0.6695 | 33.756 |
| Jazz vs. Rock | 0.6651 | 32.000 |
| Punk vs. HipHop/Rap | 0.6455 | 31.600 |
| Electronic vs. Punk | 0.6250 | 34.711 |
| Country vs. Punk | 0.5674 | 15.511 |
| Electronic vs. Rock | 0.4954 | 34.044 |
| Punk vs. Rock | 0.4876 | 57.467 |
| Jazz vs. Gospel | 0.4660 | 38.467 |
| Pop vs. HipHop/Rap | 0.4639 | 43.822 |
| Folk vs. Gospel | 0.4468 | 45.756 |
| Pop vs. Rock | 0.4048 | 56.311 |
| Jazz vs. Classical | 0.3768 | 36.889 |
| Folk vs. Country | 0.3091 | 64.933 |
