# Supplementary material for: The Aesthetic Self. The Importance of Aesthetic Taste in Music and Art for Our Perceived Identity
Source: Front Psychol. 2021 Mar 9;11:577703. doi: 10.3389/fpsyg.2020.577703 (PMC7985158; doi:10.3389/fpsyg.2020.577703)
Supplement: Supplementary file 4 [file Table_4.DOCX]

# C: Fingerhut, Gomez-Lavin, Winklmayr, Prinz: The Aesthetic Self

SUPPLEMENTARY MATERIAL C | Table with means, *SD* and Median for Self Effect for all changes in “reversed” conditions described in the Discussion section. In descending order of mean per item.

| **Category** | **Item** | **Mean (SD)** | **Median** |
| --- | --- | --- | --- |
| Beauty (reversed) | Losing one’s sense for beautiful things | 4.69 (1.512) | 5.0 |
| Music (reversed) | Not wanting to listen to music anymore | 4.31 (2.093) | 5.0 |
| Visual Art (reversed) | Not liking visual art anymore | 4.04 (1.753) | 4.0 |
| Pastime (reversed) | Losing one’s passion for video games | 3.97 (1.555) | 4.0 |
| Recreation (reversed) | Loosing one’s passion for hiking | 3.90 (1.845) | 3.5 |
